# Supplementary material for: MetaRibo-Seq measures translation in microbiomes
Source: Nat Commun. 2020 Jun 29;11:3268. doi: 10.1038/s41467-020-17081-z (PMC7324362; doi:10.1038/s41467-020-17081-z)
Supplement: Supplementary file 10 — Supplementary Data 7 [file 41467_2020_17081_MOESM10_ESM.zip › File2/Confidence_High_Taxonomy/66844_out.krona.html]

Javascript must be enabled to view this page.

members
magnitude
magnitudeUnassigned
count
unassigned
taxon
rank

66844\_out

2

2
superkingdom
2

1
phylum
976

1
117743
class

order
200644
1

family
49546
1

1
237
genus

370979
species

SRS023604\_contig\_number\_3868
1

1
phylum
1239

1737404
class
1

1
1737405
order

1570339
family
1

1
genus
162289

1465756

SRS023466\_contig\_number\_6358
species
1
